# Tumor microenvironment-responsive nanoparticles for cancer theragnostic applications

**DOI:** 10.1186/s40824-018-0132-z

**Published:** 2018-08-23

**Authors:** Saji Uthaman, Kang Moo Huh, In-Kyu Park

**Affiliations:** 1 Department of Polymer Science and Engineering, Chungnam National University, 99 Daehak-ro, Yuseong-gu, Daejeon, 34134 Republic of Korea; 2 Department of Biomedical Sciences, BK21 PLUS Centre for Creative Biomedical Scientists, Chonnam National University Medical School, 160 Baekseo-ro, Gwangju, 61469 Republic of Korea

**Keywords:** Cancer, Nanoparticle, Tumor microenvironment, pH, Redox, Hypoxia

## Abstract

**Background:**

Cancer is one of the deadliest threats to human health. Abnormal physiochemical conditions and dysregulated biosynthetic intermediates in the tumor microenvironment (TME) play a significant role in modulating cancer cells to evade or defend conventional anti-cancer therapy such as surgery, chemotherapy and radiotherapy. One of the most important challenges in the development of anti-tumor therapy is the successful delivery of therapeutic and imaging agents specifically to solid tumors.

**Main body:**

The recent progresses in development of TME responsive nanoparticles offers promising strategies for combating cancer by making use of the common attributes of tumor such as acidic and hypoxic microenvironments. In this review, we discussed the prominent strategies utilized in the development of tumor microenvironment-responsive nanoparticles and mode of release of therapeutic cargo.

**Conclusion:**

Tumor microenvironment-responsive nanoparticles offers a universal approach for anti-cancer therapy.

## Background

Cancer is one of the leading causes of mortality worldwide. Chemotherapy is one of the clinically practiced treatments for cancer. Over the past few decades, efforts have been made to deliver of small-molecule anticancer drugs to solid tumor however, therapeutic efficacy of these drugs are limited by many factors including low bio-availability, poor water solubility and poor targeting to tumor region [1]. The introduction of nanotechnology for cancer treatment has prompted the development of various nanomedicines, which are more effective and safer than conventional cancer therapies [2]. In spite of extensive research on developing tumor targeted nanomedicine, many tumors are still characterized by poor diagnosis and high mortality [3].

A major challenge faced by these cancer nanomedicines is their efficient delivery to the target solid tumors [4]. The systemic delivery of nanoparticles to the tumor site used in nanomedicine is mainly based on “active” and “passive” mechanisms [5]. Nanoparticles with long systemic circulation properties tend to accumulate in the tumor interstitial space through a passive mechanism, where selective accumulation is mainly achieved by an enhanced permeability and retention (EPR) effect and is highly dependent on the leaky vasculature and impaired lymphatics intrinsic in fast-growing tumors. In active mode, the periphery of the nanoparticles is conjugated or decorated with molecular ligands such as antibodies, peptides, biological proteins and cell-specific ligands, which may enhance the cellular uptake of nanoparticles through receptor-mediated endocytosis [6]. The active targeting of nanoparticles with targeting ligands leads to increased drug accumulation at the target tumor site, but the actual effect is limited by various tumor microenvironmental factors such as tumor heterogeneity, hypoxia and endosomal escape [7].

In recent decades, various stimuli-responsive polymers and nanoparticles that can exhibit a dramatic change in physicochemical properties in response to environmental factors, such as pH, temperature, light, reduction/oxidation, enzymes, have been designed and are now often utilized for targeted drug delivery technology. In addition to enhanced accumulation in the tumor sites mediated by active and passive targeting mechanisms, stimuli-responsive nanoparticles can facilitate augmented drug release, efficient and uniform distribution of therapeutic drug throughout the tumor and enhanced cellular uptake in response to the tumor microenvironment (TME) [6].

**Scheme 1:**
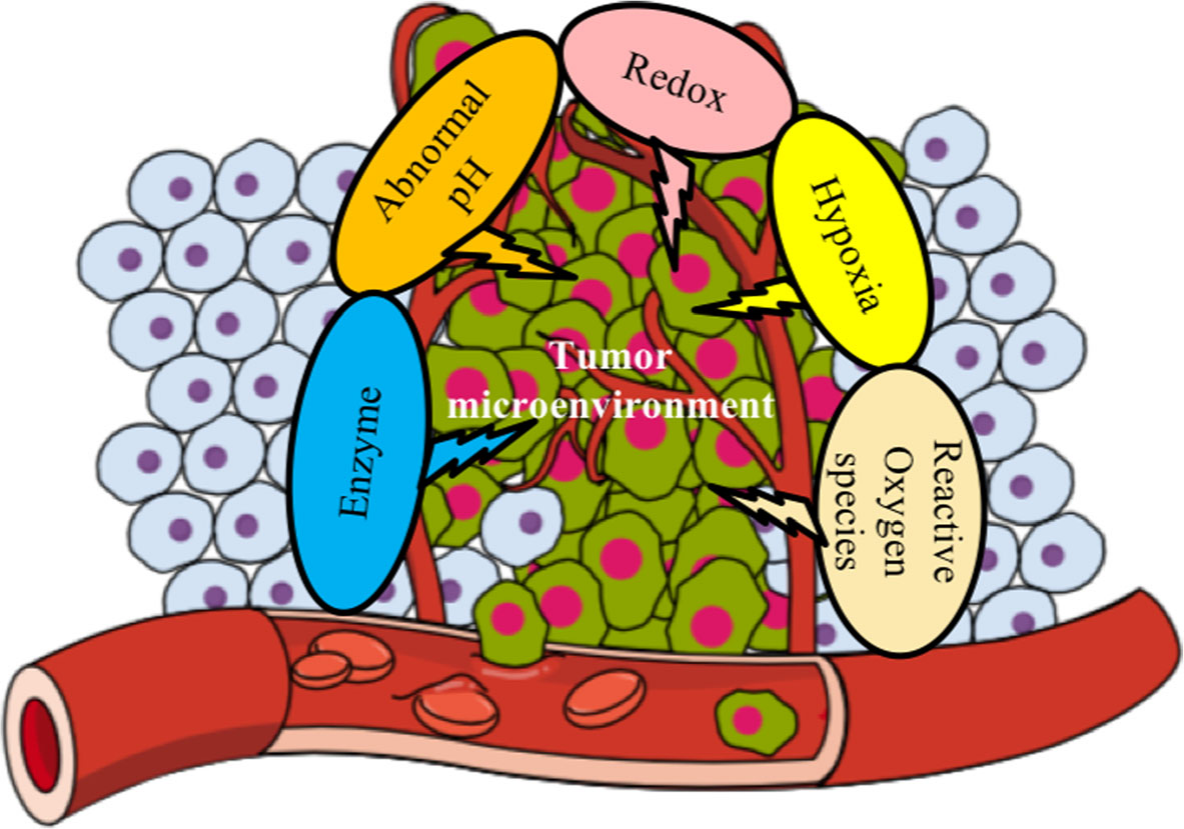
Summary of unique characteristics of TME used to develop TME-responsive nanoparticles

Compared to normal tissue, TME possesses several unique characteristics, such as acidic pH [8–12], hypoxia [6, 13–16], and higher levels of certain enzymes [17–20]. Compared to traditional nanoparticles that rely on active and passive mechanisms for tumor targeting, TME-responsive nanoparticles have several advantages. Active targeting depends on the specific interaction of a targeting moiety and/or ligands with surface receptors present on the cancer cells. The distribution and density of these receptors varies among cancer cell populations, which thus limits the broader applicability of these nanoparticles. TME-responsive nanoparticles depend on the general physiological features found in all tumors, thus offering a universal approach for anti-cancer therapy such as the site-specific release of anti-cancer drugs via TME-associated abnormal pH, hypoxia, enzymes, the redox environment and reactive oxygen species (ROS). This review describes the current status of TME-responsive nanoparticles and their functional mechanisms as exploited for targeted cancer therapy. It begins with a brief description about the common attributes of TME followed by nanoparticles activated by TME. In this review, representative examples of TME-activatable nanoparticles developed with enhanced tumor specificity and therapeutic efficacy by exploiting the unique physiological characteristics of TME (Scheme 1) are summarized.

## Targeting of common attributes of TME

As briefly mentioned above, TME possesses a variety of unique characteristics that can be utilized for the development of TME-targeted nanoparticles (Table 1). The extracellular pH in the TME is usually more acidic (pH 6.5 to pH 6.9) than the physiological pH of normal tissue (7.2 to 7.5) [21]. This acidic TME is due to the higher glycolysis rate of cancer cells to obtain the energy required for survival by converting glucose into lactic acid [6]. The pH variation in tumor cells may play an important role in designing a pH-responsive cancer targeting system. Another unique characteristic is hypoxia, wherein cells residing deep in the tumor mass are deprived of oxygen [22] due to irregular vasculature networks inside the solid tumor [23, 24]. The cells in these hypoxic regions proliferate more slowly than well-oxygenated cells, and these slow-growing cells are less susceptible to conventional anti-proliferating drugs. In addition to pH and hypoxia, the tumor microenvironment also shows altered expression of certain enzymes within tumors, which could be utilized for the TME-specific release of therapeutics [6]. Most enzymes overexpressed in the TME are from the protease family, such as membrane metalloproteinases (MMP), or from the lipase family, such as phospholipase A2 [25–27]. The specificity of enzymes to their substrates has led to the development of enzyme-responsive nanomaterials, with potential application to targeted delivery. Tumor cells in the TME experience increased potential in terms of oxidative stresses due to elevated levels of superoxide anion radicals, hydroxyl radicals and hydrogen peroxide [28]. To overcome this oxidative stress, tumor cells usually upregulate reduction potential by expressing redox species such as superoxide dismutase (SOD) and glutathione (GSH). Due to the upregulated redox level in tumors, the overall potential (oxidative/reductive) in TME is high. This dysregulation of oxidation and reduction potentials in TME makes them excellent candidates for designing TME-targeted nanoparticles. In addition, cancer cells possess elevated levels of reactive oxygen species (ROS) compared to normal cells because of the aerobic metabolism caused by oncogenic transformation [29]. All these endogenous TME stimuli offer a great opportunity for the development of TME-activatable nanoparticles.

**Table 1:** Typical examples of tumor microenvironment-responsive nanoparticles

Nanoparticle type	TME stimuli	Functionalities	Reference
Heparosan- and deoxycholic acid-conjugated micelle	Redox	GSH-responsive drug release and degradation	[52]
Gold nanoparticles	pH and Redox	Drug release controlled by pH and disassembly mediated by GSH	[63]
His-tagged fluorescent fusion protein chimera and NiFe2O4-based magnetic nanoparticles	Enzyme	MMP-2 enzyme cleavable peptide linker	[47]
Polyethyleneimine (PEI) conjugated alkylated 2-nitroimidazole (NI) and hyaluronic acid (HA) conjugated chlorin e6 (Ce6)	Hypoxia	Light and hypoxia triggered release of anti-cancer drug	[43]
Human serum Albumin nanoparticle	pH/H_2_O_2_	pH-dependent degradation of nanoparticles into smaller polymer-drug conjugates	[61]
Hollow mesoporous titanium dioxide nanoparticles	Hypoxia	Hypoxic microenvironment creation via ultrasound irradiation and hypoxia-triggered release of anti-cancer drug release of drug by hypoxia	[64]
Gold nanocluster	pH	pH-sensitive drug release	[8]
Methoxy (polyethylene glycol) thioketal-poly(ε-caprolactone) (mPEG-TK-PCL) micelles	Reactive oxygen species (ROS)	ROS-responsive drug release	[57]

### Nanoparticle activation by TME-associated abnormal pH

**Fig. 1:**
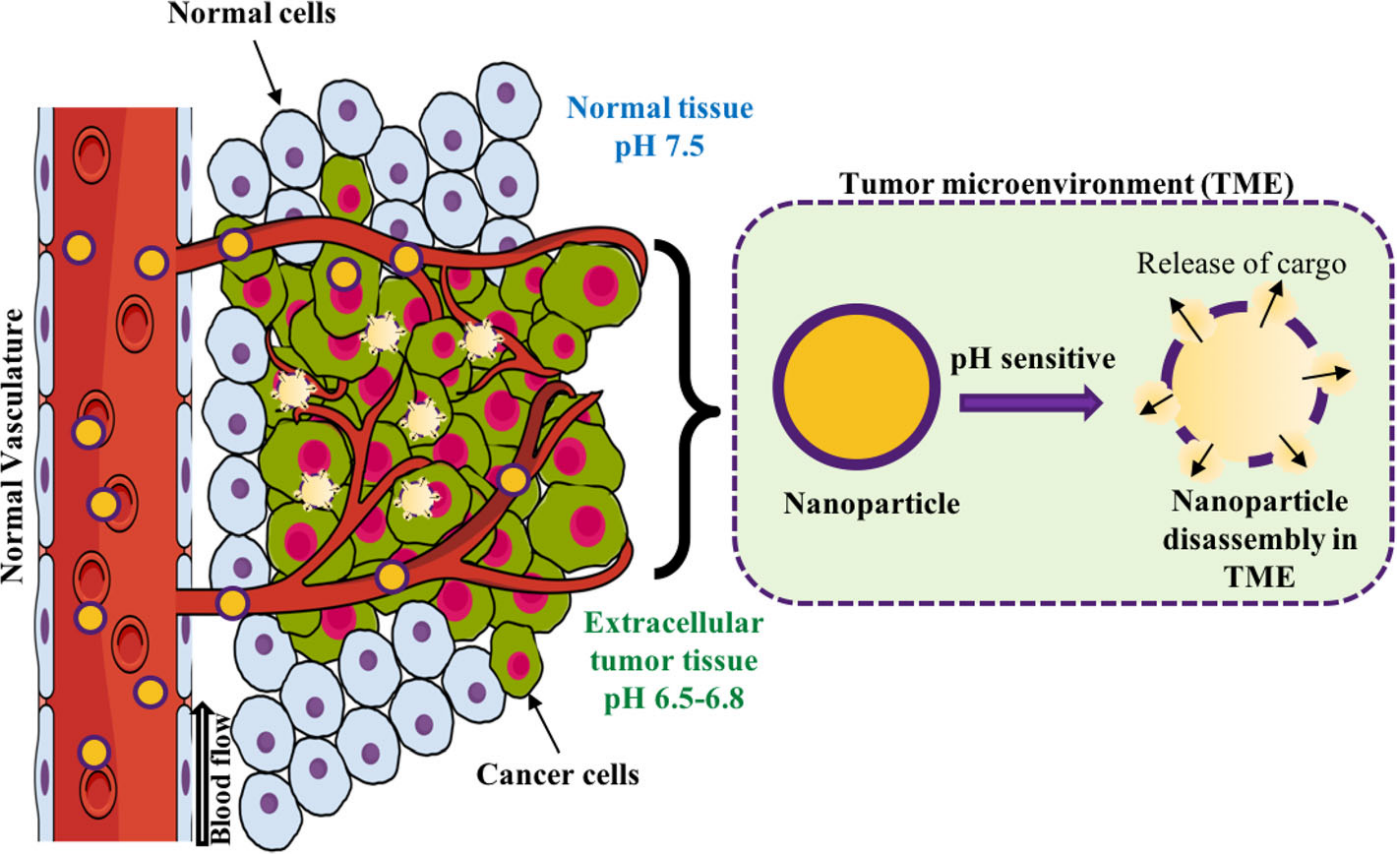
Schematic illustration of pH activation of nanoparticle by tumor microenvironment

A variety of pH-sensitive nanoparticles have been designed in recent decades and have characteristic functionalities in the molecular structure, where pKa values are close to the tumor interstitial pH. When these nanoparticles reach tumors where the microenvironmental pH is slightly acidic, a pH-dependent structural transformation occurs. The acidic environment at the tumor site triggers the protonation of pH-sensitive moieties, thereby disrupting the hydrophilic-hydrophobic equilibrium within the nanoparticle, in turn causing structural transformation and the release of therapeutic cargo loaded inside (Fig. 1). Generally, pH-responsive nanoparticles are fabricated either using acid-sensitive linkers or ionizable groups [30].

Poly(histidine) (pHis) is an attractive candidate that has been extensively used for the fabrication of a pH-sensitive drug delivery system. The pH-dependent property of pHis is due to the presence of lone-pair electrons on the unsaturated nitrogen in the imidazole group of pHis. Our group previously reported a variety of pHis-based polymeric micelles for the delivery of doxorubicin (DOX) [30–33]. Poly (ethylene glycol) methyl ether acrylate-block poly(L-lysine)-block-poly(L-histidine) triblock co-polypeptides were synthesized for pH-responsive drug delivery. The nanoparticles were found to be stable at physiological pH (7.4) but were dramatically destabilized in acidic pH due to the presence of pHis blocks [33]. The pH-induced destabilization of the nanoparticle enables the controlled release of DOX, followed by a dose-dependent cytotoxicity in murine cancer cells.

Nanoparticles have also been designed to demonstrate a pH-dependent change in surface charge. One of the most commonly investigated systems is based on zwitterionic polymers, as they have cationic and anionic groups that control surface charge in response to pH. In acidic pH, these zwitterionic polymers have a positive charge, and in basic pH, they have a negative charge. However, when these zwitterionic polymers are in neutral pH, they are overall neutral with balanced populations of positive and negative components and they become more hydrophobic. However, upon entering tumor cells, the balance between positive and negative charges will be broken and thereby cause conformational changes, facilitating drug release in tumor cells. Kang et al. [34] have reported the fabrication of tumor microenvironment responsive theragnostic with a pH-dependent fluorescence turn on/off property. The nanoparticles were constructed by encapsulating a photothermal dye (IR 825) in the carbonized zwitterionic polymer. Before accumulating in the tumor site, these nanoparticles displayed quenching of fluorescence due to the hydrophobic interaction with neutral pH and π-π stacking. The slight change in the pH in TME enabled the charge of the nanoparticles to be altered, leading to the release of IR 825 and recovered fluorescence. These types of nanoparticles can simultaneously be used for diagnosis and photothermal therapy.

pH-responsive nanoparticles have also been developed by conjugating nanocarriers with acid-labile linkage such as hydrazone [35, 36], orthoester [37, 38], imine [39, 40], phosphoramidate [41], whose hydrolysis ensures rapid the release of the drug. Liao et al. [42] have reported the synthesis of tumor targeting and pH-responsive nanoparticles for the enhanced delivery of DOX. The nanoparticles were prepared through the covalent bonding of DOX to hyaluronic acid (HA) backbone by hydrazone linkage. In aqueous solution, hyaluronic acid-hydrazone linkage-doxorubicin (HA-hyd-DOX) could self-assemble into nanoparticles. Active targeting of the nanoparticles was achieved through receptor-mediated binding of HA to CD 44, which are overexpressed in most cancer cells. These types of polymeric prodrugs could selectively release the drug in response to changes in pH. One of the major drawbacks of pH responsive nanoparticles is non-responsiveness of pH-responsive nanoparticles in the perivascular region because the acidic pH need for responsiveness is found in region far from the blood vessels. Moreover, the difference in pH between normal and tumor tissues are not significant enough for generating the responsiveness.

### Nanoparticles activation by hypoxia

**Fig. 2:**
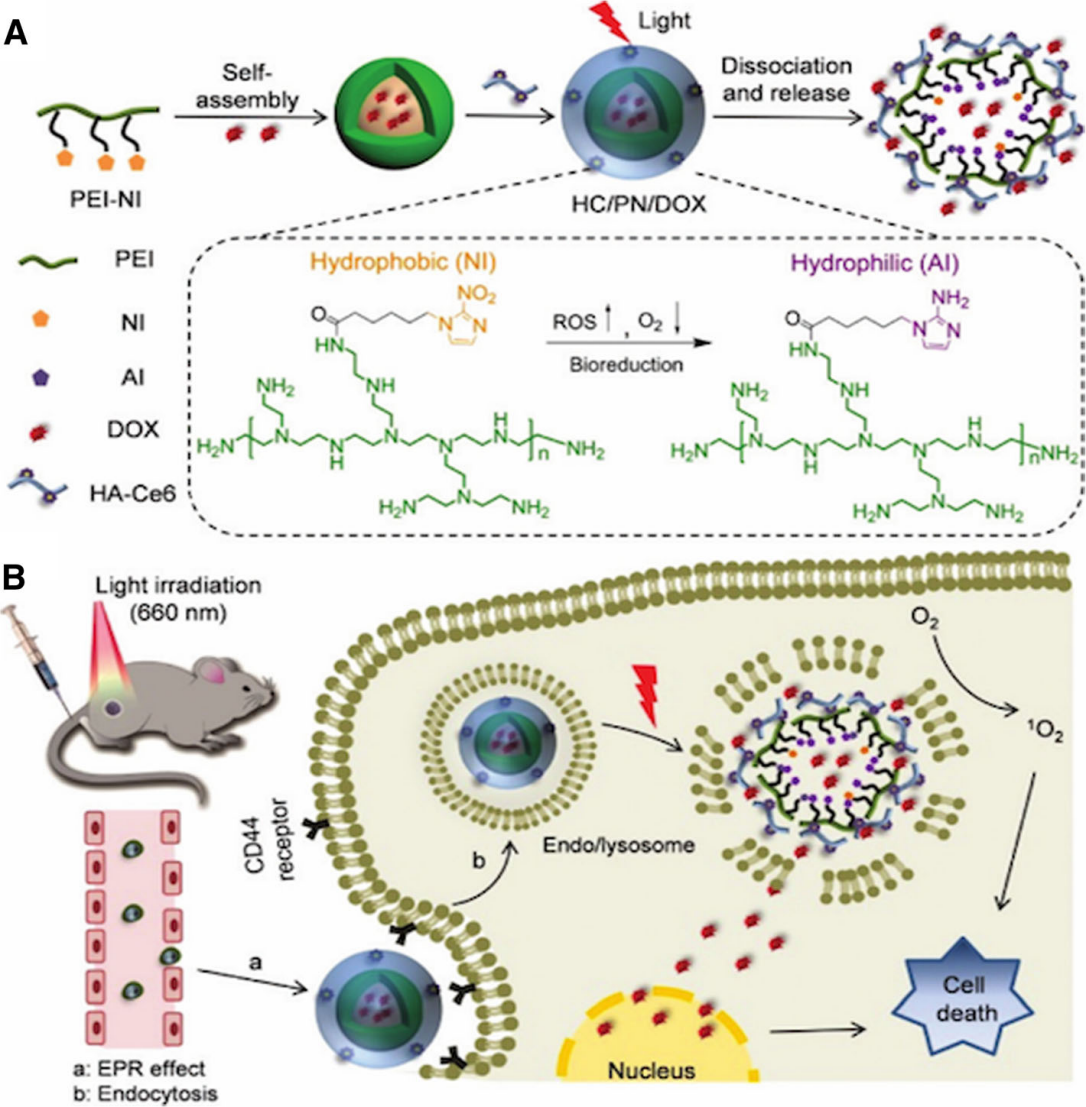
Schematic illustration of hypoxia-responsive drug delivery. **a** Fabrication of DOX-loaded PEI-NI-based nanoparticle co-assembled with HA-Ce6, (**b**) CD 44-mediated endocytosis and release of DOX in response to hypoxia generated by laser irradiation. Reproduced with permission [65] of The Royal Society of Chemistry

Due to the central role of hypoxia in enhancing tumor angiogenesis, metastasis, epithelial to mesenchymal transition, tumor invasiveness and suppression of immune reactivity [23], there has arisen great interest in the development of nanoparticles that can target the hypoxic regions within the tumor. For example, He et al. [43] reported the fabrication of dual-sensitive nanoparticles with hypoxia and photo-triggered release of the anticancer drug (Fig. 2). The authors developed dual-stimuli nanoparticles through the self-assembly of polyethyleneimine-nitroimidazole micelles (PEI-NI) further co-assembled with Ce6-linked hyaluronic acid (HC). Hypoxia-mediated activation was achieved by the incorporation of nitroimidazole (NI), a hypoxia-responsive electron acceptor. Hydrophobic NI segments would be converted to hydrophilic 2-aminoimidazole under hypoxic conditions, thereby aiding in the release of the anticancer drug (doxorubicin, DOX) loaded inside the nanoparticles.

Another hypoxia-sensitive moiety is the azobenzene group. The azobenzene (AZO) group was introduced between the polyethylene glycol (PEG) and PEI for the construction of nanocarrier for the delivery of siRNA [44]. When these particles entered into hypoxic TME, the azobenzene bond was cleaved to trigger de-shielding of the PEG coating and the subsequent release of PEI/siRNA nanoparticles. The exposed positive charge on the particles further facilitated the enhanced cellular uptake of PEI/siRNA nanoparticles. Xie et al. [45] reported the development of hypoxia responsive nanoparticles for the codelivery of siRNA and DOX. In this study, polyamidoamine (PAMAM) dendrimer was conjugated to PEG using AZO, which is a hypoxia-sensitive linker to form PAMAM-AZO-PEG (PAP). DOX was loaded into the hydrophobic core of PAMAM, and hypoxia-inducible factor 1a (HIF-1a) siRNA was electrostatically loaded onto the surface of PAMAM through ionic interactions between the anionic siRNA and amine groups of PAMAM. The PEG in PAP would prevent the nanoparticles from opsonization and prolong their circulation time in the blood. Upon reaching the tumor and exposure to hypoxic TME, PEG groups would be detached from the PAMAM surface due to the breakage of the AZO group to amino aromatics, causing the exposure of positively charged PAMAM. Once PAMAM has been taken up by tumor cells, PAMAM escapes from endosomes through the proton pump effect and releases the DOX and HIF-1a siRNA.

Yang et al. [46] have reported one pot synthesis of hollow silica nanoparticles encapsulated with catalase (CAT) and Ce6 doped into the silica lattice. CAT is a water soluble H_2_O_2_ decomposing enzyme which triggers the decomposition of H_2_O_2_ to H_2_O and O_2_. The nanoparticles were further modified with mitochondrial targeting moiety ((3-carboxypropyl) triphenyl phosphonium bromide (CTPP)) and pH responsive charge convertible polymer through electrostatic interaction. Upon reaching acidic tumor microenvironment, the polymeric coating would undergo charge conversion from negative to positive, thereby enhancing the cellular internalization. The mitochondrial targeting moiety helps in enhancing photodynamic therapy induced cell death and the catalase encapsulated inside would decompose the tumor endogenous H_2_O_2_, thereby overcoming hypoxic environment in the tumor and enhancing the photodynamic therapy of solid tumors. These types of smart nanoparticles can overcome the limitations of conventional photodynamic therapy. Despite the advances in the development of hypoxia responsive nanoparticles, getting these nanoparticles into hypoxic region is quite challenging as these regions are typically located deep inside the tumor with less vasculature, where the mass transport is through diffusion. For most of the nanoparticle systems, the diffusion rate would be insufficient within solid tumors and hence nanocarriers with higher diffusion rate of small molecules would be a better option for carrying and releasing hypoxia-activated prodrugs within TME.

### Nanoparticles activation by enzymes

**Fig. 3:**
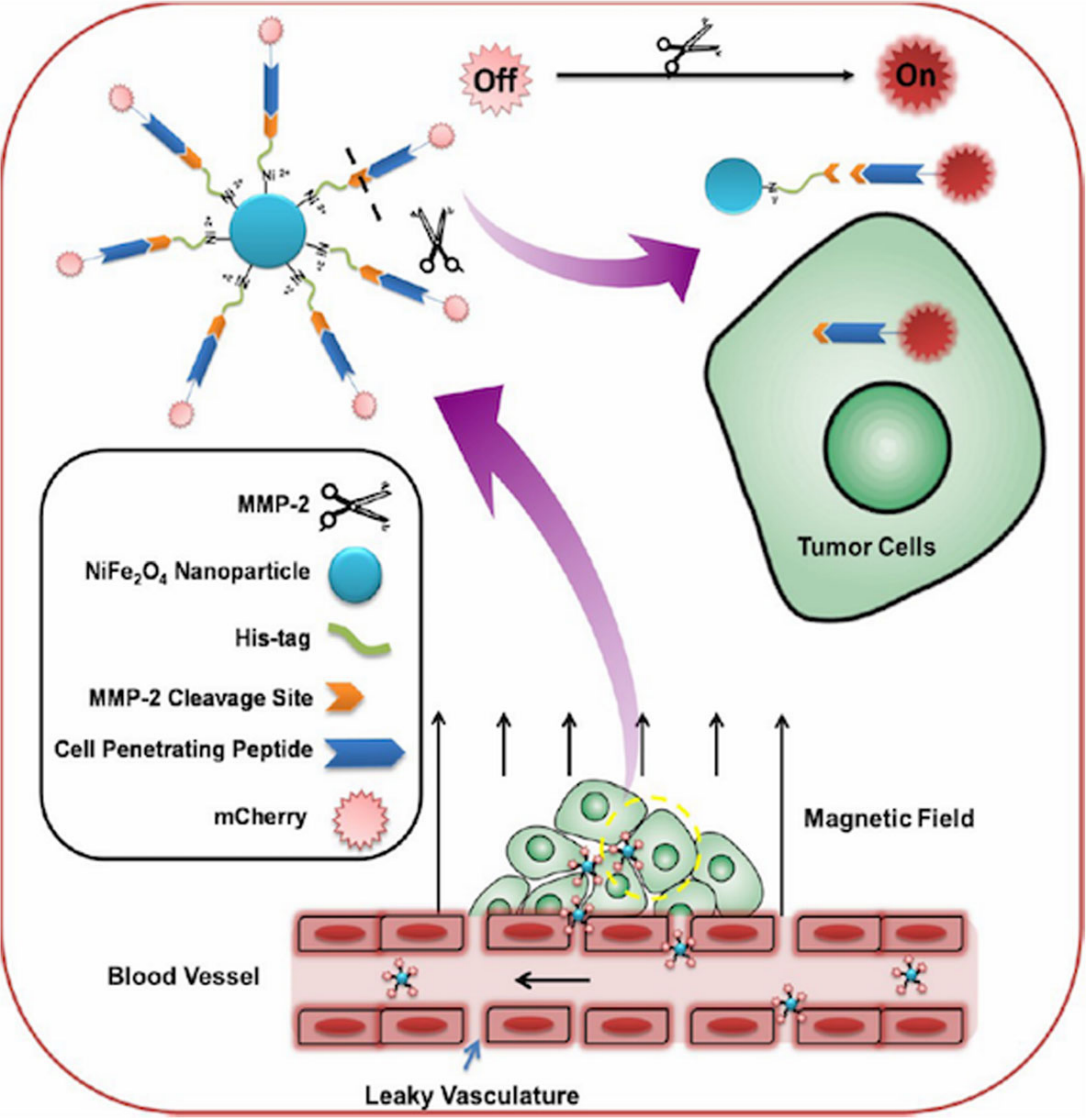
Schematic illustration of enzyme-responsive magnetic nanoprobe. Reproduced with permission [48] Copyright © 2017, American Chemical Society

TME also have upregulated levels of enzymes such as matrix metalloproteinase (MMP), which is predominantly involved in tumor development and proliferation [19]. The upregulated levels of MMP enzymes in TME makes them the most common target for enzyme-based TME nanoparticles. Sun et al. [47] reported the development of MMP-2 activatable nanoprobes, which can be used for selective and specific intracellular imaging of the tumor (Fig. 3). The nanoprobe was constructed through the self-assembly of hexahistidine-tagged (His-Tagged) fluorescent protein and nickel ferrite nanoparticles. The nickel ferrite nanoparticles functioned as protein binders of His-Tagged fluorescent protein and fluorescent quencher. The nanoprobe was reported to be turned on by the presence of MMP-2, leading to enhanced cellular uptake and the restoration of fluorescence, thereby enabling the visualization of nanoparticles within tumor tissue. Ma et al. [48] reported the fabrication of polymeric conjugate for mitochondrial targeting for paclitaxel (PTX) delivery. The polymeric conjugate consists of a PAMAM-based dendrimer core into which triphenylphosphine and PTX were conjugated through an amino bond and disulfide bonds, respectively. To enhance the circulation time of the polymeric conjugate in the blood, PEG was conjugated via the MMP-2 sensitive peptide (GPLGIAGQ). The conjugates accumulate in tumor tissue through the EPR effect. Once the conjugate enters tumor cells, the PEG layer is detached from PAMAM by cleavage of MMP-2 sensitive peptide by the action of MMP-2. The conjugate would then target the mitochondria via triphenylphosphine and PTX would be released in the cytoplasm.

Ansari et al. [49] have reported the synthesis of theranostic nanoparticles which possess enzyme specific drug release and in vivo magnetic resonance imaging (MRI). The nanoparticles were synthesized through the conjugation of ferumoxytol (FDA approved iron oxide nanoparticles) to MMP-14 activatable peptide conjugated to azademetylcolchicine (ICT) (CLIO-ICT). Upon reaching tumor the CLIO-ICT would be converted from non-toxic form to toxic form by the action of MMP-14 thereby, releasing potent ICT. This type of nanoparticles also enables the real-time monitoring of accumulation and localization of drug at the tumor site through MRI imaging.

Another type of enzyme whose levels are known to be upregulated in various cancer subtypes is β-galactosidase (β-gal) [50]. Sharma et al. [50] have developed theragnostic prodrug for the treatment of colon cancer using receptor mediated targeting and enzyme responsive activation. In this study β-gal was used for both targeting asialoglycoprotein (ASGP) receptors and activation of prodrug. When delivered these nanoparticles would be preferentially taken up by the colon cancer cells through receptor mediated endocytosis and the anti-cancer drug will be released by the enzymatic activation. One of the major concerns in enzyme responsive therapy is the heterogenous expression of the target enzyme in different types of cancer and difference in the level of target enzyme at different stages of cancer. To develop more effective and precise enzyme responsive delivery vehicles, more better understanding of the spatial and temporal patterns of enzyme at target site is needed.

### Nanoparticle-activated by redox environment

The intracellular GSH level inside TME are in the range of 0.5–10 × 10^− 3^ M, which is four times higher than the GSH levels in normal tissues [28]. Intracellular compartments such as the cytosol, mitochondria, and cell nucleus are known to contain a much higher concentration of GSH than extracellular fluids. Such drastic differences in the GSH level between TME and other normal tissue could be utilized as a promising platform to design nanoparticles to selectively release therapeutic drugs in a triggered fashion after delivery to the tumor cells [32, 51]. The introduction of bio-reducible disulfide bonds has attracted much interest in the design of redox-responsive nanoparticles that can release their payloads efficiently in intracellular reductive environments.

**Fig. 4:**
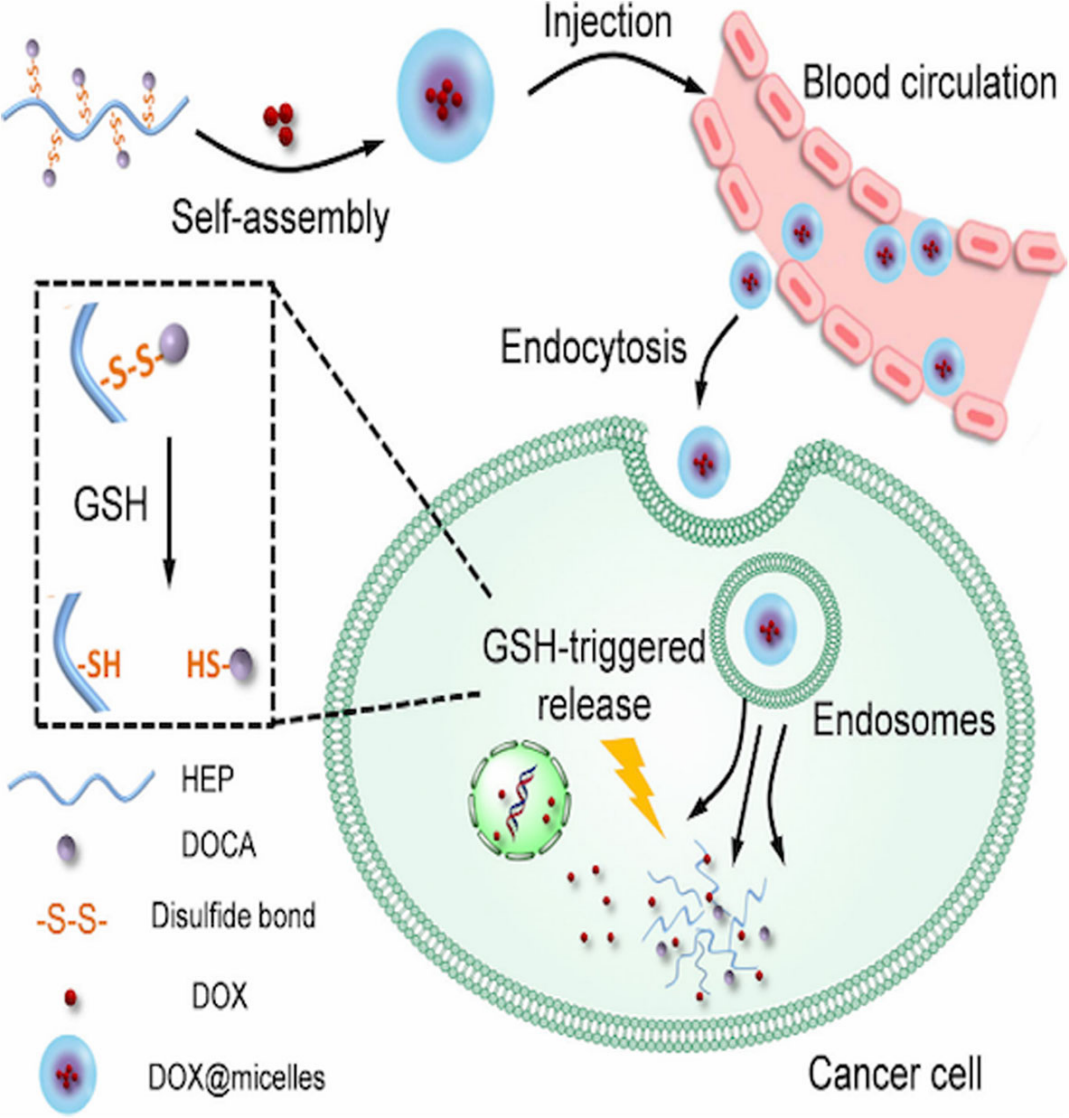
Schematic illustration of self-assembled micelle and GSH triggered release of DOX. Reproduced with permission [52] Copyright © 2018, Elsevier

Sun et al. [52] have reported the synthesis of a redox-sensitive drug delivery system for the treatment of laryngopharyngeal carcinoma (Fig. 4). The redox-sensitive amphiphilic polymer was synthesized by conjugating heparosan with deoxycholic acid through disulfide bonding. The polymer formed self-assembled nanoparticles that can disassemble via reductive cleavage of the di-sulfide bonds and trigger drug release in the intracellular environment. Our group has also reported the synthesis of zwitterionic polymer-based hybrid nanoparticles with glutathione and endosomal pH-responsiveness [31, 32]. GSH-responsive drug delivery systems could selectively deliver the drug in TME and enhance the antitumor efficacy of the nanoparticle.

Zhou et al. [53] have reported the synthesis of redox sensitive drug delivery system based on dextran and indomethacin. Redox responsive polymer (DEX-SS-IND) was fabricated through the introduction of di-sulfide bridge (cystamine) in between based on dextran and indomethacin. Anti-cancer drug, DOX was encapsulated inside the core-shelled micelles formed by self-assembly of DEX-SS-IND. In reducing environment, the DEX-SS-IND depolymerizes and releases DOX. In-vivo antitumor efficacy of DOX loaded DEX-SS-IND micelles were more compared to DOX loaded non-redox responsive polymer. Xia et al. [54] have reported the synthesis of polycarbonate-based core-crosslinked redox responsive nanoparticles (CC-RRNs) for the targeted delivery of DOX. CC-RRNs were synthesized by the click reaction between PEG-b-poly (MPC)n (PMPC), α-lipoic acid and 6-bromohexanoic acid. The di-sulfide cross linked core is formed by the addition of catalytic amount of dithiothreitol (DTT). CC-RRNs demonstrated controlled release of DOX under redox condition. Such multifunctional responsive systems hold the key for future developments in TME-assisted nanomedicine. However, it would be noted that exact intracellular fate of redox sensitive nanoparticles is not clearly understood. Studies have reported that cell surface thiols can affect the internalization of di-sulfide conjugated peptides [55]. Hence, a better understanding about the intracellular trafficking of the nanoparticles is required for development of nanoparticle-activated by redox environment.

### Reactive oxygen species (ROS) responsive nanoparticles

In cancer cells, the level of ROS is higher than in normal cells, due to the constant production of ROS as the byproducts of aerobic metabolism caused by oncogenic transformation [29]. This higher level of ROS in tumors could be utilized for the development of ROS-responsive nanoparticles, which could enhance site-specific drug release. The most commonly used characteristic groups employed for the development of ROS-responsive systems are boronic ester [56], thioketal [57] and sulfide [58] groups. Such ROS-responsive systems can lead to the development of drug carriers for efficient delivery of chemotherapy.

**Fig. 5:**
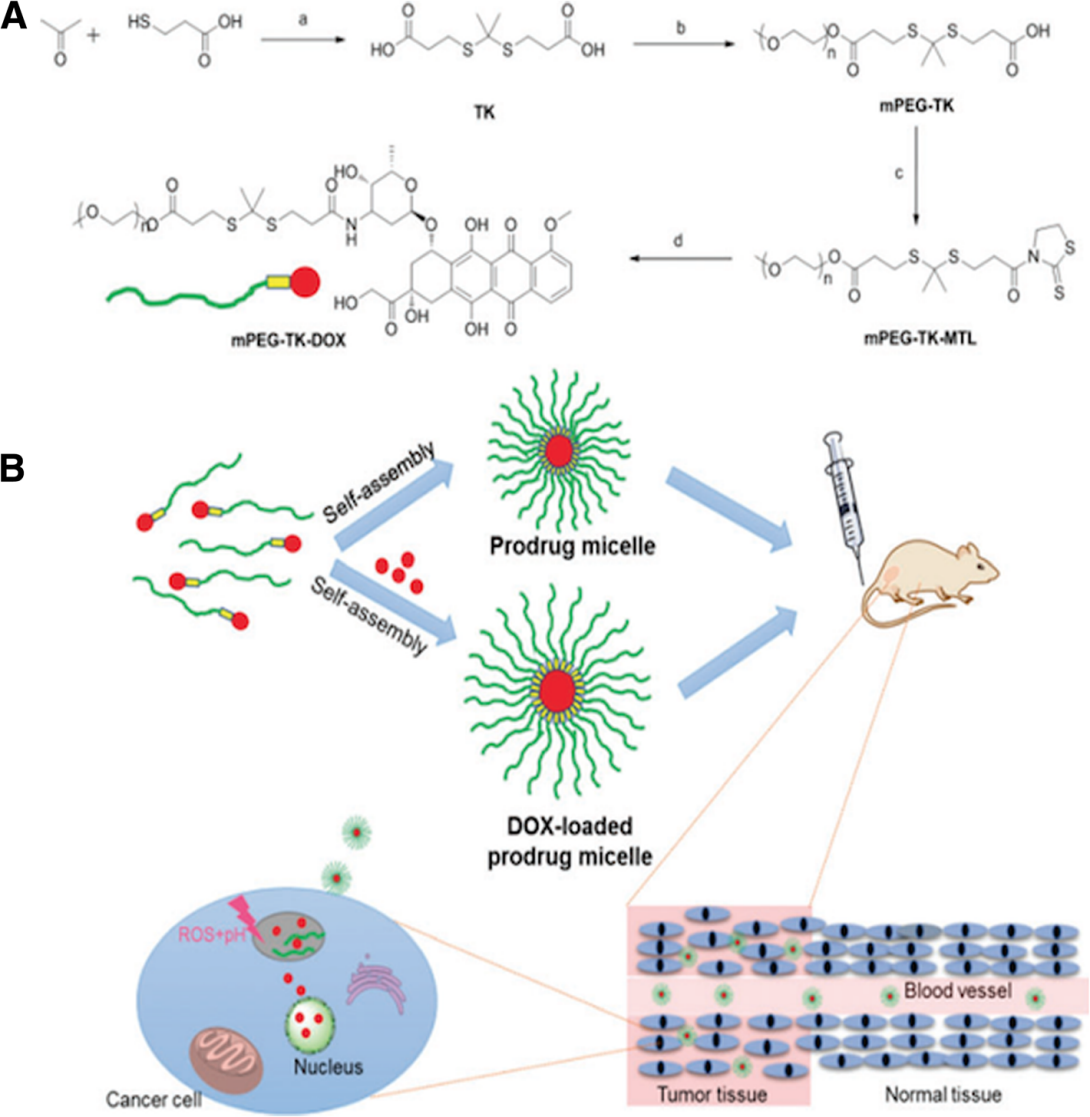
Schematic illustration of (**a**) synthesis of ROS–responsive prodrug mPEG-TK- DOX and (**b**) illustration of ROS–responsive prodrug micelle and DOX-loaded prodrug micelle for drug delivery. Reproduced with permission [1] Copyright © 2018, Royal Society of Chemistry

Sun et al. [57] have developed ROS-responsive micelles for enhanced drug delivery applications. For the development of ROS-responsive micelles, a ROS-sensitive thioketal linker with a π-conjugated structure was conjugated into methoxy (polyethylene glycol) thioketal-poly(ε-caprolactone) (mPEG-TK-PCL) micelles. The micelles were formed through the self- assembly of mPEG-TK-PCL and DOX was then loaded through physical encapsulation. The DOX-loaded mPEG-TK-PCL micelles demonstrated enhanced anticancer activity due to the rapid cleavage of the thioketal linker in the presence of increased ROS levels in cancer cells, thereby accelerating drug release and augmenting cancer cell inhibition. Xu et al. [1] have developed a ROS-responsive prodrug through the thioketal linkage of PEG and DOX (Fig. 5). The prodrug was then used as a drug carrier to further encapsulate DOX and form DOX-loaded prodrug micelles. DOX-loaded prodrug micelles demonstrated superior anti-tumor efficacy over non-responsive DOX-loaded poly (ethylene glycol)-block-polycaprolactone (PEG2k-PCL5k) micelles.

Yu et al. [59] have reported the synthesis of chalcogen containing polycarbonate for ROS responsive PDT. The ROS responsive polycarbonate was prepared by the ring opening polymerization of cyclic carbonate monomers with ethyl selenide, phenyl selenide or ethyl telluride group. PEG was employed as macro-initiator to prepare amphiphilic block co-polymers, which forms spherical nanoparticles of less than 100 nm. These nanoparticles completely dissociate in the presence of ROS while remain stable in neutral phosphate buffer. To check the ROS responsive drug release potential of these nanoparticles, DOX and Ce6 were loaded. Upon laser irradiation, Ce6 would generate ^1^O_2_ which will trigger the degradation of the nanoparticle resulting in the faster release of DOX. Even though numerous ROS responsive nanoparticles have been reported for biomedical application, there are several challenges needed to be addressed such as the biocompatibility of the ROS sensitive linker used, stability of the linker during circulation and at the normal cells. Since the levels of ROS changes with variations in patients and disease conditions the selection of linkers and carrier should be intensively considered for personalized application.

### Multi-stimuli responsive nanoparticles

To obtain greater specificity and efficacy, the various stimuli responsive drug delivery system discussed above were often used in combinations. Xiong et al. [60] have reported the synthesis of pH/redox sensitive micelles for the delivery of DOX and gold nanoparticles (GNPs). The micelles comprise of an amphiphilic copolymer of poly(ε-caprolactone)-ss-poly(2-(dimethylamino) ethyl methacrylate) (PCL-SS-PDMAEMA). The PDMAEMA will protonate in acidic conditions, thereby enhancing the hydrophilicity and swelling of the micellar shell and di-sulfide bond will be cleaved when exposed to abundance of GSH, thereby causing the disassembly of the micellular structure. DOX was loaded in the hydrophobic PCL core and GNPs in the hydrophilic PDMAEMA region. GNPs work as a contrast agent for tumor imaging and diagnosis through computed tomography (CT). The core shell micelles showed better drug release in tumor cells by pH triggered swelling and GSH triggered disassembly.

Chen et al. [61] have demonstrated pH /H_2_O_2_ responsive nanoparticles to modulate tumor hypoxia. In this study, human serum albumin (HSA) was pre-modified with either photosensitizer chlorine e6 (Ce6) or with pro-drug of cisplatin and then the HSA was used as a template for formation of manganese dioxide (MnO_2_). Under acidic condition, MnO_2_ would decompose and reactive with H_2_O_2_ to produce O_2_, which will help in overcoming the tumor hypoxia-associated resistance to PDT. Upon intravenous injection, the nanoparticles accumulate in tumor region through EPR effect and then degrade into smaller HAS complexes which possess better intra-tumor penetration ability.

Jia et al. [62] have reported the synthesis of redox/enzyme responsive nitric oxide (NO) releasing nanoparticles for anti-cancer therapy. The nanoparticle comprises of organic-inorganic composite and encapsulate glutathione S-transferases π (GSTπ)- responsive drug O2-(2,4-dinitro-5- {[2-(β d-galactopyranosylolean-12-en-28-oate-3-yl)-oxy-2-oxoethyl] piperazine-1-yl} phenyl)1-(methylethanolamino) diazen-1-ium-1,2-dilate (NPQ) as NO donor. The nanoparticle demonstrated responsiveness towards the GSH, resulting in the biodegradation of shell of the nanoparticle, aiding in the release of NPQ and subsequently NO within the tumor.

Conclusion and Perspectives.

In recent decades, various tumor-targeting technologies have developed with a compromise between efficacy and safety. The effort to design nanoparticles that can selectively accumulate at tumor sites by passive and active targeting mechanisms has improved cancer treatment with limited success. Recent advances in the development of TME targeted nanoparticle-based therapy have been summarized in this review. TME-targeted nanoparticle-based therapies exploit the unique characteristics of TME, such as acidic pH, hypoxia, redox species, upregulated levels of enzymes and reactive oxygen levels. To further develop nanoparticles with higher theragnostic performance with minimal harmful side effects due to anti-cancer therapy, a combination of TME targeted nanoparticles and with immunotherapy would be beneficial.
